# Parametrized inequality of Hermite-Hadamard type for functions whose third derivative absolute values are quasi-convex

**DOI:** 10.1186/s40064-015-1633-z

**Published:** 2015-12-30

**Authors:** Shan-He Wu, Banyat Sroysang, Jin-Shan Xie, Yu-Ming Chu

**Affiliations:** Department of Mathematics, Longyan University, Longyan, 364012 Fujian People’s Republic of China; Department of Mathematics and Statistics, Faculty of Science and Technology, Thammasat University, Pathumthani, 12121 Thailand; School of Mathematics and Computation Science, Hunan City University, Yiyang, 413000 Hunan People’s Republic of China

**Keywords:** Hermite-Hadamard type inequality, Parameter, Quasi-convex, Special means, 26D10, 26A51, 26E60

## Abstract

In this paper we present some inequalities of Hermite-Hadamard type for functions whose third derivative absolute values are quasi-convex. Moreover, an application to special means of real numbers is also considered.

## Background

A real-valued function *f* defined on an interval $I\subseteq \mathbb {R}$ is said to be convex on *I*, if

$$\begin{aligned} f(\lambda x + (1-\lambda )y) \le \lambda f(x) + (1-\lambda )f(y) \end{aligned}$$

for all $x,y\in I$ and $\lambda \in [0,1]$.

If *f* is convex on *I*, then we have the Hermite-Hadamard inequality (see Mitrinović et al. 1993)

1 $$\begin{aligned} f\left( \frac{a+b}{2}\right) \le \frac{1}{b-a}\displaystyle \int _{a}^{b}f(x) dx \le \frac{f(a)+f(b)}{2} \end{aligned}$$

for all $$a,\,b\in I$$.

A function $f: I\subseteq \mathbb {R}\rightarrow \mathbb {R}$ is said to be quasi-convex on *I*, if

$$\begin{aligned} f(\lambda x + (1-\lambda )y) \le \max \left\{ f(x),f(y)\right\} \end{aligned}$$

for all $x,y\in I$ and $\lambda \in [0,1]$.

Clearly, any convex function is a quasi-convex function. Furthermore, there exist quasi-convex functions which are not convex.

In 2007, Ion (2007) presented an inequality of Hermite-Hadamard type for functions whose derivatives in absolute values are quasi-convex functions, as follows:

### **Theorem 1.1**

*Letf* : $I\subset \mathbb {R} \rightarrow \mathbb {R}$* be a differentiable mapping on*$I^{o}$, $a, b\in I$* with*$a<b$.* If *$\;\left| f{^{\prime }}\right|$* is quasi-convex on* [*a*, *b*],* then the following inequality holds*:

2 $$\begin{aligned} \left| \frac{f(a)+f(b)}{2} - \frac{1}{b-a}\int _{a}^{b}f(x) dx\right| \le \frac{b-a}{4}\max \left\{ \left| f{^{\prime }}(a)\right| ,\left| f{^{\prime }}(b)\right| \right\} . \end{aligned}$$

In 2010, Alomari et al. (2010a) established an analogous version of inequality (1), which is asserted by Theorem 1.2 below:

### **Theorem 1.2**

*Let f* : $I\subset \mathbb {R} \rightarrow \mathbb {R}$* be twice differentiable mapping on *$I^{o}$, $a, b\in I$* with *$a<b$* and *$f^{{\prime\prime }}$* is integrable on* [*a*, *b*].* If*$\;\left| f^{{\prime\prime }}\right|$* is quasi-convex on* [*a*, *b*],* then the following inequality holds:*

3 $$\begin{aligned} \displaystyle \left| \frac{f(a)+f(b)}{2} - \frac{1}{b-a}\int _{a}^{b}f(x) dx\right| \le \frac{(b-a)^2}{12}\max \left\{ \left| f^{{\prime\prime }}(a)\right| ,\left| f^{{\prime\prime }}(b)\right| \right\} . \end{aligned}$$

Recently, Guo et al. (2015) investigated Hermite-Hadamard type inequalities for geometrically quasi-convex functions. Xi and Qi (2014, 2015) and Xi et al. (2012, 2014) showed some new Hermite-Hadamard type inequalities for s-convex functions. For more results relating to refinements, counterparts, generalizations of Hadamard type inequalities, we refer interested readers to Alomari et al. (2010b), Chen (2015), Niculescu and Persson (2006), Pečarić et al. (1992), Sroysang (2014), Sroysang (2013) and Wu (2009).

The main purpose of this paper is to present a parametrized inequality of Hermite-Hadamard type for functions whose third derivative absolute values are quasi-convex. As applications, some new inequalities for special means of real numbers are established.

## Lemmas

In order to prove our main results, we need the following lemmas.

### **Lemma 2.1**

*Let *$\epsilon \in \mathbb {R}$* and let f*: $I\subset \mathbb {R}\rightarrow \mathbb {R}$* be three times differentiable on*$I^{\circ }$* and *$\; a,b \in I$* with*$a<b$.* Assume that *$f^{{\prime\prime\prime}}$* is integrable on *[*a*, *b*]. Then

$$\begin{aligned} &\frac{(4-\epsilon )f(a)+(2+\epsilon )f(b)}{6} - \frac{1}{b-a}\int _{a}^{b}f(x) dx - \frac{b-a}{12}\left( \epsilon f{^{\prime }}(b) - (2-\epsilon )f{^{\prime }}(a) \right) \\ &\quad = \frac{(b-a)^3}{12} \int _{0}^{1} \lambda (1-\lambda )(2\lambda -\epsilon ) f^{{\prime\prime\prime }}(\lambda a + (1-\lambda )b)d\lambda . \end{aligned}$$

### *Proof*

Integrating by parts, we have

$$\begin{aligned} \displaystyle&\int _{0}^{1} \lambda (1-\lambda )(2\lambda -\epsilon ) f^{{\prime\prime\prime }}(\lambda a + (1-\lambda )b)d\lambda \\&\quad = \frac{1}{a-b} \int _{\lambda =0}^{\lambda =1} \lambda (1-\lambda )(2\lambda -\epsilon ) d f^{{\prime\prime }}(\lambda a + (1-\lambda )b)\\&\quad = \frac{1}{a-b} \left[ \lambda (1-\lambda )(2\lambda -\epsilon ) f^{{\prime\prime }}(\lambda a + (1-\lambda )b)\right] _{\lambda =0}^{\lambda =1}\\&\quad \quad - \frac{1}{a-b} \int _{\lambda =0}^{\lambda =1} f^{{\prime\prime }}(\lambda a + (1-\lambda )b) d\left( \lambda (1-\lambda )(2\lambda -\epsilon )\right) \\&\quad = - \frac{1}{a-b} \int _{\lambda =0}^{\lambda =1} f^{{\prime\prime }}(\lambda a + (1-\lambda )b) d\left( \lambda (1-\lambda )(2\lambda -\epsilon )\right) \\&\quad = \frac{1}{b-a} \int _{\lambda =0}^{\lambda =1} \left( -6\lambda ^2 + 2(2+\epsilon )\lambda -\epsilon \right) f^{{\prime\prime }}(\lambda a + (1-\lambda )b) d\lambda \\&\quad = \frac{1}{(b-a)^2} \int _{\lambda =0}^{\lambda =1} \left( 6\lambda ^2 - 2(2+\epsilon )\lambda +\epsilon \right) d f{^{\prime }}(\lambda a + (1-\lambda )b) \\&\quad = \frac{1}{(b-a)^2} \left[ \left( 6\lambda ^2 - 2(2+\epsilon )\lambda +\epsilon \right) f{^{\prime }}(\lambda a + (1-\lambda )b)\right] _{\lambda =0}^{\lambda =1} \\&\quad \quad - \frac{1}{(b-a)^2} \int _{\lambda =0}^{\lambda =1} f{^{\prime }}(\lambda a + (1-\lambda )b) d\left( 6\lambda ^2 - 2(2+\epsilon )\lambda +\epsilon \right) \\&\quad = \frac{-1}{(b-a)^2} \left( \epsilon f{^{\prime }}(b) - (2-\epsilon )f{^{\prime }}(a) \right) \\&\quad \quad - \frac{1}{(b-a)^2} \int _{\lambda =0}^{\lambda =1}\left( 12\lambda - 2(2+\epsilon ) \right) f{^{\prime }}(\lambda a + (1-\lambda )b) d\lambda \\&\quad = \frac{-1}{(b-a)^2} \left( \epsilon f{^{\prime }}(b) - (2-\epsilon )f{^{\prime }}(a) \right) \\&\quad \quad + \frac{2}{(b-a)^3} \int _{\lambda =0}^{\lambda =1} ( 6\lambda - 2-\epsilon ) d f(\lambda a + (1-\lambda )b) \\&\quad = \frac{-1}{(b-a)^2} \left( \epsilon f{^{\prime }}(b) - (2-\epsilon )f{^{\prime }}(a) \right) \\&\quad \quad + \frac{2}{(b-a)^3} \left[ ( 6\lambda - 2-\epsilon )f(\lambda a + (1-\lambda )b) \right] _{\lambda =0}^{\lambda =1} \\&\quad \quad - \frac{2}{(b-a)^3} \int _{\lambda =0}^{\lambda =1} f(\lambda a + (1-\lambda )b) d( 6\lambda - 2-\epsilon ) \\&\quad = \frac{-1}{(b-a)^2} \left( \epsilon f{^{\prime }}(b) - (2-\epsilon )f{^{\prime }}(a) \right) \\&\quad \quad + \frac{2}{(b-a)^3} \left( (4-\epsilon )f(a)+(2+\epsilon )f(b) \right) \\&\quad \quad - \frac{12}{(b-a)^3} \int _{\lambda =0}^{\lambda =1} f(\lambda a + (1-\lambda )b) d\lambda . \end{aligned}$$

Changing variable $x = \lambda a + (1-\lambda )b$, it follows that

$$\begin{aligned} \displaystyle&\int _{0}^{1} \lambda (1-\lambda )(2\lambda -\epsilon ) f{^{{\prime\prime\prime }}}(\lambda a + (1-\lambda )b)d\lambda \\&\quad = \frac{-1}{(b-a)^2} \left( \epsilon f{^{\prime }}(b) - (2-\epsilon )f{^{\prime }}(a) \right) \\&\quad \quad + \frac{2}{(b-a)^3} \left( (4-\epsilon )f(a)+(2+\epsilon )f(b) \right) \\&\quad \quad - \frac{12}{(b-a)^4} \int _{x=a}^{x=b} f(x) dx. \end{aligned}$$

Thus,

$$\begin{aligned} \displaystyle&\frac{(b-a)^3}{12} \int _{0}^{1} \lambda (1-\lambda )(2\lambda -\epsilon ) f{^{{\prime\prime\prime }}}(\lambda a + (1-\lambda )b)d\lambda \\&\quad = \frac{(4-\epsilon )f(a)+(2+\epsilon )f(b)}{6} - \frac{1}{b-a}\int _{a}^{b}f(x) dx - \frac{b-a}{12}\left( \epsilon f{^{\prime }}(b) - (2-\epsilon )f{^{\prime }}(a) \right) . \end{aligned}$$

The proof of Lemma 2.1 is completed. $\square$

### **Lemma 2.2**

*Let*$\epsilon$* be a real number. Then*

$$\begin{aligned} \displaystyle \int _{0}^{1} \lambda (1-\lambda ) \left| 2\lambda -\epsilon \right| d\lambda = {\left\{ \begin{array}{ll} \displaystyle \frac{\epsilon - 1}{6} &{}\quad \text{ if } \epsilon \ge 2 \\ \displaystyle \frac{4\epsilon ^3 - \epsilon ^4}{48} + \frac{1-\epsilon }{6} &{}\quad \text{ if } 0 < \epsilon < 2 \\ \displaystyle \frac{1-\epsilon }{6} &{}\quad \text{ if } \epsilon \le 0. \end{array}\right. } \end{aligned}$$

### *Proof*

We distinguish three cases

**Case 1** If $\epsilon \ge 2$, then

$$\begin{aligned} \displaystyle \int _{0}^{1} \lambda (1-\lambda ) \left| 2\lambda -\epsilon \right| d\lambda = \int _{0}^{1} \lambda (1-\lambda ) (\epsilon -2\lambda ) d\lambda = \frac{\epsilon - 1}{6}. \end{aligned}$$

**Case 2** If $0 < \epsilon < 2$, then

$$\begin{aligned} \displaystyle \int _{0}^{1} \lambda (1-\lambda ) \left| 2\lambda -\epsilon \right| d\lambda&= \int _{0}^{\epsilon /2} \lambda (1-\lambda ) (\epsilon -2\lambda ) d\lambda + \int _{\epsilon /2}^{1} \lambda (1-\lambda ) (2\lambda -\epsilon ) d\lambda \\&= \frac{4\epsilon ^3 - \epsilon ^4}{48} + \frac{1-\epsilon }{6}. \end{aligned}$$

**Case 3** If $\epsilon \le 0$, then

$$\begin{aligned} \displaystyle \int _{0}^{1} \lambda (1-\lambda ) \left| 2\lambda -\epsilon \right| d\lambda = \int _{0}^{1} \lambda (1-\lambda ) (2\lambda -\epsilon ) d\lambda = \frac{1-\epsilon }{6}. \end{aligned}$$

This completes the proof of Lemma 2.2. $\square$

## Main results

Our main results are stated in the following theorems.

### **Theorem 3.1**

*Let*$q\ge 1$* and*$\epsilon \in \mathbb {R}$,* and let f* : $I\subset \mathbb {R}\rightarrow \mathbb {R}$* be three times differentiable on*$I^{\circ }$* and *$\; a,b \in I$* with*$a<b$.* Assume that*$f{^{{\prime\prime\prime }}}$* is integrable on* [*a*, *b*],* and*$\left| f{^{{\prime\prime\prime }}}\right| ^q$* is quasi-convex on* [*a*, *b*].* Then*

$$\begin{aligned} \displaystyle&\left| \frac{(4-\epsilon )f(a)+(2+\epsilon )f(b)}{6} - \frac{1}{b-a}\int _{a}^{b}f(x) dx - \frac{b-a}{12}\left( \epsilon f{^{\prime }}(b) - (2-\epsilon )f{^{\prime }}(a) \right) \right| \\&\\&\quad \le {\left\{ \begin{array}{ll} \displaystyle \frac{(b-a)^3}{12}\left( \frac{\epsilon - 1}{6}\right) \left( \max \left\{ \left| f{^{{\prime\prime\prime }}}(a)\right| ^q,\left| f{^{{\prime\prime\prime}}}(b)\right| ^q\right\} \right) ^{1/q} &{}\quad \text{ if } \epsilon \ge 2 \\ \displaystyle \frac{(b-a)^3}{12}\left( \frac{4\epsilon ^3 - \epsilon ^4}{48} + \frac{1-\epsilon }{6}\right) \left( \max \left\{ \left| f{^{{\prime\prime\prime}}}(a)\right| ^q,\left| f{^{{\prime\prime\prime}}}(b)\right| ^q\right\} \right) ^{1/q} &{}\quad \text{ if } 0 < \epsilon < 2 \\ \displaystyle \frac{(b-a)^3}{12} \left( \frac{1-\epsilon }{6}\right) \left( \max \left\{ \left| f{^{{\prime\prime\prime}}}(a)\right| ^q,\left| f{^{{\prime\prime\prime}}}(b)\right| ^q\right\} \right) ^{1/q} &{} \quad \text{ if } \epsilon \le 0. \end{array}\right. } \end{aligned}$$

### *Proof*

Using Lemma 2.1 and Hölder’s inequality gives

$$\begin{aligned} \displaystyle&\left| \frac{(4-\epsilon )f(a)+(2+\epsilon )f(b)}{6} - \frac{1}{b-a}\int _{a}^{b}f(x) dx - \frac{b-a}{12}\left( \epsilon f{^{\prime }}(b) - (2-\epsilon )f{^{\prime }}(a) \right) \right| \\&\quad \le \frac{(b-a)^3}{12} \int _{0}^{1} \lambda (1-\lambda )\left| 2\lambda -\epsilon \right| \left| f{^{{\prime\prime\prime}}}(\lambda a + (1-\lambda )b)\right| d\lambda \\&\quad = \frac{(b-a)^3}{12} \int _{0}^{1} \left( \lambda (1-\lambda )\left| 2\lambda -\epsilon \right| \right) ^{1-1/q} \\&\quad \quad \times \left( \lambda (1-\lambda )\left| 2\lambda -\epsilon \right| \left| f{^{{\prime\prime\prime}}}(\lambda a + (1-\lambda )b)\right| ^q\right) ^{1/q} d\lambda \\&\quad \le \frac{(b-a)^3}{12} \left( \int _{0}^{1} \lambda (1-\lambda )\left| 2\lambda -\epsilon \right| d\lambda \right) ^{1-1/q} \\&\qquad \times \left( \int _{0}^{1} \lambda (1-\lambda )\left| 2\lambda -\epsilon \right| \left| f{^{{\prime\prime\prime}}}(\lambda a + (1-\lambda )b)\right| ^q d\lambda \right) ^{1/q}. \end{aligned}$$

By the quasi-convexity of $\left| f{^{{\prime\prime\prime}}}\right| ^q$, we obtain

$$\begin{aligned} \displaystyle&\left| \frac{(4-\epsilon )f(a)+(2+\epsilon )f(b)}{6} - \frac{1}{b-a}\int _{a}^{b}f(x) dx - \frac{b-a}{12}\left( \epsilon f{^{\prime }}(b) - (2-\epsilon )f{^{\prime }}(a) \right) \right| \\&\quad \le \frac{(b-a)^3}{12} \left( \int _{0}^{1} \lambda (1-\lambda )\left| 2\lambda -\epsilon \right| d\lambda \right) ^{1-1/q} \\&\quad \times \left( \int _{0}^{1} \lambda (1-\lambda )\left| 2\lambda -\epsilon \right| \max \left\{ \left| f{^{{\prime\prime\prime}}}(a)\right| ^q,\left| f{^{{\prime\prime\prime}}}(b)\right| ^q\right\} d\lambda \right) ^{1/q} \\&\quad = \frac{(b-a)^3}{12} \left( \int _{0}^{1} \lambda (1-\lambda )\left| 2\lambda -\epsilon \right| d\lambda \right) \left( \max \left\{ \left| f{^{{\prime\prime\prime}}}(a)\right| ^q,\left| f{^{{\prime\prime\prime}}}(b)\right| ^q\right\} \right) ^{1/q}. \end{aligned}$$

Utilizing Lemma 2.2 leads to the desired inequality in Theorem 3.1. $\square$

### *Remark 3.2*

It is worth noticing that if we use a substitution $a \rightarrow b$, $b\rightarrow a$ and $\epsilon \rightarrow 2-\epsilon$ in Theorem 3.1, we have the following further generalization of Theorem 3.1.

### **Theorem 3.3**

*Let*$q\ge 1$* and*$\epsilon \in \mathbb {R}$,* and let f* : $I\subset \mathbb {R}\rightarrow \mathbb {R}$* be three times differentiable on *$I^{\circ }$, $\; a,b \in I$* with *$a\ne b$.* Assume that *$f{^{{\prime\prime\prime}}}$* is integrable on* [*a*, *b*],* and*$\left| f{^{{\prime\prime\prime}}}\right| ^q$* is quasi-convex on the closed interval formed by the points a* and *b*.* Then*

$$\begin{aligned} \displaystyle&\left| \frac{(4-\epsilon )f(a)+(2+\epsilon )f(b)}{6} - \frac{1}{b-a}\int _{a}^{b}f(x) dx - \frac{b-a}{12}\left( \epsilon f{^{\prime }}(b) - (2-\epsilon )f{^{\prime }}(a) \right) \right| \\&\\&\quad \le {\left\{ \begin{array}{ll} \displaystyle \frac{|b-a|^3}{12}\left( \frac{\epsilon - 1}{6}\right) \left( \max \left\{ \left| f{^{{\prime\prime\prime }}}(a)\right| ^q,\left| f{^{{\prime\prime\prime}}}(b)\right| ^q\right\} \right) ^{1/q} &{}\quad \text{ if } \epsilon \ge 2 \\ \displaystyle \frac{|b-a|^3}{12}\left( \frac{4\epsilon ^3 - \epsilon ^4}{48} + \frac{1-\epsilon }{6}\right) \left( \max \left\{ \left| f{^{{\prime\prime\prime}}}(a)\right| ^q,\left| f{^{{\prime\prime\prime}}}(b)\right| ^q\right\} \right) ^{1/q} &{}\quad \text{ if } 0 < \epsilon < 2 \\ \displaystyle \frac{|b-a|^3}{12} \left( \frac{1-\epsilon }{6}\right) \left( \max \left\{ \left| f{^{{\prime\prime\prime}}}(a)\right| ^q,\left| f{^{{\prime\prime\prime}}}(b)\right| ^q\right\} \right) ^{1/q} &{} \quad \text{ if } \epsilon \le 0. \end{array}\right. } \end{aligned}$$

As a direct consequence, choosing $\displaystyle \epsilon = 1$ in Theorem 3.3, we get the following inequality:

### **Corollary 3.4**

*Let *$q\ge 1$* and *$\epsilon \in \mathbb {R}$,* and letf* : $I\subset \mathbb {R}\rightarrow \mathbb {R}$* be three times differentiable on*$I^{\circ }$, $\; a,b \in I$* with*$a\ne b$.* Assume that *$f{^{{\prime }{\prime }{\prime}}}$* is integrable on* [*a*, *b*], and $\left| f{^{{\prime\prime\prime}}}\right| ^q$* is quasi-convex on the closed interval formed by the pointsa and b*.* Then*

$$\begin{aligned} \displaystyle&\left| \frac{f(a)+f(b)}{2} - \frac{1}{b-a}\int _{a}^{b}f(x) dx - \frac{b-a}{12}\left( f{^{\prime }}(b) - f{^{\prime }}(a) \right) \right| \\&\quad \le \frac{|b-a|^3}{192}\left( \max \left\{ \left| f{^{{\prime\prime\prime}}}(a)\right| ^q,\left| f{^{{\prime\prime\prime}}}(b)\right| ^q\right\} \right) ^{1/q}. \end{aligned}$$

In addition, if we utilize Theorem 3.1 with a substitution of $$\;\epsilon = 0,\;0.5,$$$$\;3,\;-2,\;-3,\;-5,$$ respectively, then we obtain the following results:

### **Corollary 3.5**

*Let*$q\ge 1$* and *$\epsilon \in \mathbb {R}$,* and let f* : $I\subset \mathbb {R}\rightarrow \mathbb {R}$* be three times differentiable on*$I^{\circ }$, $\; a,b \in I$* with*$a\ne b$.* Assume that *$f{^{{\prime\prime\prime}}}$* is integrable on* [*a*, *b*], and $\left| f{^{{\prime\prime\prime}}}\right| ^q$* is quasi-convex on the closed interval formed by the pointsa* and *b*.* Then*

$$\begin{aligned} \displaystyle&\left| \frac{2f(a)+f(b)}{3} - \frac{1}{b-a}\int _{a}^{b}f(x) dx + \frac{b-a}{6}f{^{\prime }}(a)\right| \\&\quad \le \frac{|b-a|^3}{72}\left( \max \left\{ \left| f{^{{\prime\prime\prime}}}(a)\right| ^q,\left| f{^{{\prime\prime\prime}}}(b)\right| ^q\right\} \right) ^{1/q}, \end{aligned}$$

$$\begin{aligned} \displaystyle&\left| \frac{7f(a)+5f(b)}{12} - \frac{1}{b-a}\int _{a}^{b}f(x) dx - \frac{b-a}{24}\left( f{^{\prime }}(b) - 3f{^{\prime }}(a) \right) \right| \\&\quad \le \frac{71|b-a|^3}{9216}\left( \max \left\{ \left| f{^{{\prime\prime\prime}}}(a)\right| ^q,\left| f{^{{\prime\prime\prime}}}(b)\right| ^q\right\} \right) ^{1/q}, \end{aligned}$$

$$\begin{aligned} \displaystyle&\left| \frac{f(a)+5f(b)}{6} - \frac{1}{b-a}\int _{a}^{b}f(x) dx - \frac{b-a}{12}\left( 3f{^{\prime }}(b) + f{^{\prime }}(a) \right) \right| \\&\quad \le \frac{|b-a|^3}{36}\left( \max \left\{ \left| f{^{{\prime\prime\prime}}}(a)\right| ^q,\left| f{^{{\prime\prime\prime}}}(b)\right| ^q\right\} \right) ^{1/q}, \end{aligned}$$

$$\begin{aligned} \displaystyle&\left| f(a) - \frac{1}{b-a}\int _{a}^{b}f(x) dx + \frac{b-a}{6}\left( f{^{\prime }}(b) + 2f{^{\prime }}(a) \right) \right| \\&\quad \le \frac{|b-a|^3}{24}\left( \max \left\{ \left| f{^{{\prime\prime\prime}}}(a)\right| ^q,\left| f{^{{\prime\prime\prime}}}(b)\right| ^q\right\} \right) ^{1/q}, \end{aligned}$$

$$\begin{aligned} \displaystyle&\left| \frac{7f(a)-f(b)}{6} - \frac{1}{b-a}\int _{a}^{b}f(x) dx + \frac{b-a}{12}\left( 3f{^{\prime }}(b) + 5f{^{\prime }}(a) \right) \right| \\&\quad \le \frac{|b-a|^3}{18}\left( \max \left\{ \left| f{^{{\prime\prime\prime}}}(a)\right| ^q,\left| f{^{{\prime\prime\prime}}}(b)\right| ^q\right\} \right) ^{1/q}, \end{aligned}$$

$$\begin{aligned} \displaystyle&\left| \frac{3f(a)-f(b)}{2} - \frac{1}{b-a}\int _{a}^{b}f(x) dx + \frac{b-a}{12}\left( 5f{^{\prime }}(b) + 7f{^{\prime }}(a) \right) \right| \\&\quad \le \frac{|b-a|^3}{12}\left( \max \left\{ \left| f{^{{\prime\prime\prime}}}(a)\right| ^q,\left| f{^{{\prime\prime\prime}}}(b)\right| ^q\right\} \right) ^{1/q}. \end{aligned}$$

## Applications to special means

We now consider the applications of our results to the special means of real numbers.

The weighted arithmetic mean of real numbers $\left\{ a,b\right\}$ with weight $\left\{ w_a,w_b\right\}$ is defined by

$$\begin{aligned} A(a,b;w_a,w_b) = \frac{w_a a + w_b b}{w_a+w_b}, \end{aligned}$$

  where $$ a,b, w_a, w_b \in \mathbb {R}$$ with $$w_a+w_b\ne 0$$.

In particularly

$$\begin{aligned} A(a,b;1,1) = A(a,b)=\frac{a +b}{2}, \end{aligned}$$

  which is called the arithmetic means.

The generalized logarithmic mean of real numbers $\left\{ a,b\right\}$ is defined by

$$\begin{aligned} L_{n}(a,b) = \left[ \frac{ b^{n+1}- a^{n+1}}{(n+1)(b-a)}\right] ^\frac{1}{n}, \end{aligned}$$

where $$ a,b,\in \mathbb {R}$$, $$n \in \mathbb {Z}$$ with $$n\ne 0, -1$$, $$a\ne b$$.

### **Proposition 4.1**

*Let*$a,b,\epsilon \in \mathbb {R}$, $a\ne b$, $\epsilon \ne 1$* and*$n \in \mathbb {N}, n\ge 3$.* Then, we have*

$$\begin{aligned} \displaystyle&\left| A(a^{n},b^{n};4-\epsilon ,2+\epsilon ) -\frac{(b-a)(\epsilon -1)n}{6}A(a^{{n-1}},b^{{n-1}};\epsilon -2,\epsilon )-L_{n}^{n}(a,b) \right| \\ {}&\\&\le {\left\{ \begin{array}{ll} \displaystyle \frac{|b-a|^3}{72}\left( {\epsilon - 1}\right) n(n-1)(n-2)\left( \max \left\{ |a|^{n-3},|b|^{n-3}\right\} \right) &{}\quad \text{ if } \epsilon \ge 2\\ \displaystyle \frac{|b-a|^3}{576}\left( {8-8\epsilon +4\epsilon ^3-\epsilon ^4 }\right) n(n-1)(n-2)\left( \max \left\{ |a|^{n-3},|b|^{n-3}\right\} \right) &{} \quad \text{ if } 0 < \epsilon < 2 \\ \displaystyle \frac{|b-a|^3}{72}\left( {1-\epsilon }\right) n(n-1)(n-2)\left( \max \left\{ |a|^{n-3},|b|^{n-3}\right\} \right) &{} \quad \text{ if } \epsilon \le 0. \end{array}\right. } \end{aligned}$$

### *Proof*

We consider the function $f(x)=x^n$, $x \in \mathbb {R}$, $n\ge 3$. It is easy to verify that the function $f{^{{\prime\prime\prime}}}(x)=n(n-1)(n-2)x^{n-3}$ is quasi-convex on $(-\infty ,+\infty )$ (see Alomari et al. 2010b). The assertion follows from Theorem 3.3 with $q=1$. $\square$

### *Remark 4.2*

In a similar way as the proof of the Proposition 4.1, one can easily deduce from Corollary 3.4 the following inequality.

### **Proposition 4.3**

*Let *$a,b \in \mathbb {R}$, $a\ne b$* and *$n \in \mathbb {N}, n\ge 3$.* Then, we have*

$$\begin{aligned}&\left| A(a^{n},b^{n})-\frac{(b-a)^2}{12}n(n-1)L_{n-2}^{n-2}(a,b)-L_{n}^{n}(a,b) \right| \\&\quad \le \frac{|b-a|^3}{192}n(n-1)(n-2)\left( \max \left\{ |a|^{n-3},|b|^{n-3}\right\} \right) . \end{aligned}$$

## Conclusions

This paper provides some new results related to the Hermite-Hadamard type inequalities. Firstly, we present a parametrized inequality of Hermite-Hadamard type for functions whose third derivative absolute values are quasi-convex, the main results are given in Theorems 3.1 and 3.3. As special cases, by assigning special value to the parameter, one can obtain several new and previously known results for Hermite-Hadamard type inequality (Corollaries 3.4 and 3.5). Secondly, as applications of the obtained results, we establish two new inequalities involving special means of real numbers by using the parametrized Hermite-Hadamard type inequality (see Propositions 4.1 and 4.3).
